# Evaluation of Before Operational Stress: A Program to Support Mental Health and Proactive Psychological Protection in Public Safety Personnel

**DOI:** 10.3389/fpsyg.2021.511755

**Published:** 2021-08-17

**Authors:** Andrea M. Stelnicki, Laleh Jamshidi, Amber J. Fletcher, R. Nicholas Carleton

**Affiliations:** ^1^Canadian Institute for Public Safety Research & Treatment, University of Regina, Regina, SK, Canada; ^2^Department of Sociology and Social Studies, University of Regina, Regina, SK, Canada; ^3^Anxiety and Illness Behaviours Lab, Department of Psychology, University of Regina, Regina, SK, Canada

**Keywords:** public safety personnel, operational stress injuries, mental health, Before Operational Stress, posttraumatic stress, intervention protocol, proactive psychological protection

## Abstract

Public safety personnel (PSP; e.g., communications officials, corrections workers, firefighters, paramedics, and police officers) are at risk of developing mental health problems due to experiencing potentially psychologically traumatic events during their career. Research examining evidence-based treatments for psychological injuries resulting from operational duties (also known as operational stress injuries) has not yielded robust results that would indicate ongoing interventions as the best solution for managing PSP mental health injuries; as such, proactive psychological interventions designed to bolster resilience are being considered potentially beneficial for mitigating the impact of occupational stress on PSP. Despite the growing popularity of resilience programs, most are delivered in a single session after an event deemed particularly problematic with no follow-up. Longer interventions may better support sustained resiliency, mitigate the impact of operational stress, and increase positive PSP workplace outcomes. The current article introduces the Before Operational Stress (BOS) program, which was designed for delivery early in a PSP career to enhance self-awareness and healthy relationships. The year-long program is derived from cognitive behavior therapy and group therapeutic techniques to meet program objectives. The current BOS program evaluation demonstrated small, statistically significant improvements in symptoms of PTSD, quality of life, stigma, and perceived social support from baseline (Time 1) to 6 months (Time 4). There were also non-significant improvements observed in symptoms of depression, anxiety, stress, alcohol use, as well as in emotional regulation and resilience. Qualitative results indicated participants positively perceived the BOS program, with participants reporting specific improvements in self-awareness, avoidant behaviors, and relationships with family and colleagues. The BOS program content (e.g., functional disconnection and functional reconnection) and processes (e.g., psychoeducation within a supportive learning structure; mutually empowering group interactions) appear unique relative to other PSP resilience programs, with promising initial results in support of PSP mental health. Recommendations for future research and program development are provided.

## Introduction

Public safety personnel (PSP; e.g., border services workers, communications officials, correctional workers, firefighters, paramedics, and police officers) appear to experience higher prevalence rates of mental disorders than the general population (Carleton et al., 2018a). PSP are regularly exposed to potentially psychologically traumatic events (PPTE; Canadian Institute for Public Safety Research Treatment, 2019) that can increase risk for developing psychological difficulties including posttraumatic stress disorder (PTSD), generalized anxiety disorder, depression, social anxiety disorder and panic disorder (Carleton et al., 2019a). In Canada, psychological difficulties that result from operational duties are collectively termed operational stress injuries (OSIs; Richardson et al., 2008; Oliphant, 2016; Canadian Institute for Public Safety Research Treatment, 2019). PSP can reasonably be expected to remain at elevated risk for OSIs for the duration of their careers.

Many different PPTE-focused treatments have been empirically validated and supported since PTSD was introduced in DSM-III (American Psychiatric Association, 1980). For example, cognitive behavior therapy (Foa and Rothbaum, 1998), eye movement desensitization and reprocessing (Shapiro, 1999), and prolonged exposure therapy (Foa and Kozak, 1986) have all been evidenced as helpful to many people exposed to PPTE (American Psychological Association, 2017); however, no standard of care is deemed helpful for all patients (Foa et al., 2000; Ponniah and Hollon, 2009). Treatment protocols have typically been adapted from military and veteran populations who experience PTSD because of combat exposure to support other populations (Papazoglou, 2017); however, PPTE exposures for PSP can be qualitatively different from exposures related to combat or exposures experienced by most of the general population (Oliphant, 2016; Papazoglou, 2017; McElheran et al., 2018; Carleton et al., 2019a). Research evaluating treatments for PSP has not yielded sufficient results to support evidence-based recommendations for managing symptoms that can come from repeated PPTE exposures (Haugen et al., 2012).

PSP culture can be influenced by mental health stigma, stoicism, and avoidant coping strategies (Ricciardelli et al., 2018a,b), all of which can confound self-awareness of OSI symptoms (Karaffa and Koch, 2015) and negatively impact help-seeking behaviors (Oliphant, 2016; Ricciardelli et al., 2018a,b). OSIs are often not recognized until the impacts on the PSP are significant (e.g., marital strain, interpersonal problems with coworkers, problematic substance use; Ricciardelli et al., 2018a). Asking for help can be considered a sign of weakness among PSP and may be accompanied by fears of potential consequences (e.g., being taken off the job, limits for promotions or transfers, loss of professional identity; Wester et al., 2010). Occupational stressors include both operational stressors (e.g., PPTE exposures, shift work, job-related risk of injury) and organizational stressors (e.g., perceived lack of support, inconsistent leadership, lack of resources; McCreary and Thompson, 2006). The impact of diverse occupational stressors has received more attention over the past several years, due in part to greater awareness (Carleton et al., 2018a), government engagement (Oliphant, 2016), and popularity of training programs focused on mental health [e.g., Road to Mental Readiness (R2MR) developed by the Canadian Department of National Defense; National Defence the Canadian Armed Forces, 2017]. Mental health training programs are typically designed to provide psychoeducation, minimize stigma and stereotypes, and clarify how to access additional resources as needed for mental health (Papazoglou and Andersen, 2014; Beshai and Carleton, 2016; Anderson et al., 2020); however, the evidence that proactive strategies can mitigate the impact of PPTE exposures remains far behind the evidence for treatments. Proactive strategies might be particularly important tools for maintaining the mental health of PSP who may experience thousands of PPTEs during their careers (Carleton et al., 2019b).

Programs like R2MR have been designed to improve performance, reduce barriers to care, encourage early access to care, and provide tools to manage mental disorder symptoms (National Defence the Canadian Armed Forces, 2017). There are now many adaptations and versions of the R2MR content and delivery method, but in general the content is presented as a 4-h (for frontline employees) or 8-h (for supervisors) classroom-based educational program. Early evidence for the R2MR content suggests there are associations with small reductions in stigma and small increases in resiliency skills from pre- to post-training and at three-month follow up (Szeto et al., 2019). Police officers who received R2MR were found to have small but non-significant changes to mental health symptoms, resilience, and work engagement following training (Carleton et al., 2018b). PSP who have completed R2MR training screen positive for mental disorders at a slightly lower rate than PSP with no mental health training (Carleton et al., 2019b); however, PSP with R2MR training reported a preference to access informal supports (e.g., spouse, colleague, and friend) before they access mental health professionals (e.g., psychologist, psychiatrist, or employee assistance program; Carleton et al., 2019b), suggesting single sessions of R2MR training may not be sufficient to encourage help-seeking among PSP. Training programs that involve longer interactions with participants and opportunities to integrate learned concepts into occupational activities may result in larger and sustained improvements in mental health and personal resilience (Leppin et al., 2014). For example, a 10-week resilience program that involved both classroom learning combined with practice in realistic, stressful simulations for new police officers produced significant and large improvements for negative mood, better work performance, and significant physiological improvements (e.g., less heart rate reactivity; Arnetz et al., 2009).

The recent evidence of pervasive mental health challenges among PSP coupled with clinical feedback suggests that PSP may need and want more mental health education as part of preparations for the potential impact of stress from repeated exposures to potentially traumatic events as a function of their operational duties. PSP may also need the education to be ongoing, focused on support, and provided in a culturally-competent context.

## The Before Operational Stress (BOS) Program

The Before Operational Stress (BOS) program (WGM Psychological Services, 2018) was designed to fill a gap in available PSP services. BOS program development was led by Dr. Megan McElheran of WGM Psychological Services^1^ in Calgary, AB, Canada. BOS combines educational and didactic material derived from well-established elements of cognitive behavioral therapies with group processing facilitated by a mental health clinician. The program material is provided to participants for 8 consecutive weeks (active phase), followed by 10 monthly follow-up sessions (maintenance phase). Development of the program began in 2016 following feedback from PSP indicating a broad desire for early access to cognitive behavioral therapy content and supports. The initial version of BOS was completed late in 2017 and proposed as a program to be funded by Wounded Warriors Canada (WWC) who became a charitable sponsor in early 2018.

WWC has subsidized the cost to participants of BOS as part of a 3-year pilot that includes independent program evaluation by researchers associated with the Canadian Institute for Public Safety Research and Treatment (CIPSRT). The independent evaluation is part of a transition WWC supports for all programming to move from being evidence-informed to evidence-based. There were two groups of PSP in Alberta (firefighters) and Ontario (paramedics) recruited to receive BOS training and participate in the pilot program evaluation. The group participants provided valuable feedback on the program and BOS in its current form was officially launched in October 2018.

BOS was not designed to be, and is not intended to be, a PPTE-focused treatment program. BOS was designed as a program to increase self-awareness and encourage authentic, healthy relationships specifically for PSP. Participants become more aware of spiritual (in BOS, meaning “of the self”), physical, emotional, and mental aspects of themselves during the first half of the eight-week program. The first six BOS content modules are based on cognitive behavior therapy (Beck, 1976; Beck et al., 1985), which involves teaching participants to identify, understand, and navigate the connection between thoughts, emotions, physiological sensations, and behavior. Greater self-awareness can initiate specific coping mechanisms that can be used when PSP are confronted with operational stressors, including but not limited to PPTEs. Active coping strategies are discussed within each content module. The last two BOS content modules build on elements of cognitive behavior therapy but focus on enhancing interpersonal relationships through communication skills and empathy. Spouses and friends may be particularly important for PSP mental health (Carleton et al., 2019b); as such, maintaining healthy, authentic relationships may also be particularly important. Healthy, authentic relationships depend on strong communication which in turn depends on having sufficient self-awareness (Sutton et al., 2015). Throughout all eight BOS content modules, participants are taught to develop an awareness of how and when to seek additional support.

The BOS program content focuses on the current impact of operational stressors on the individual PSP. Although organizational stressors may also be part of the PSP experience, BOS specifically focuses on how PSP react to operational stress. There are at least four aspects of BOS that are currently unique relative to other available programming. First, BOS is designed to provide substantial amounts of time for participants to receive, reflect upon, and practice the educational component within a supportive learning structure. The learning is facilitated by an interactive mental health clinician to make the information relevant and applicable to individual participants' daily experiences. Approximately half of each session is dedicated to interactive engagement tailoring the material to one or more specific participant experiences. Participants are encouraged to talk about their struggles and successes when working with the information learned in BOS. Participants are also supported in problem solving how to incorporate the BOS content into their operational roles. The interactive experiences within the BOS program are believed to be at least as significant a mechanism for change as the actual content. The image of a stereotypical, stoic PSP maintains a common assumption that PSP would be unwilling to discuss their diverse vocational challenges (e.g., occupational stressors; Ricciardelli et al., 2018a; Carleton et al., 2019b); in contrast, PSP members who have participated in BOS generally find comfort in sharing experiences within an environment of like-minded peers (M. McElheran, personal communication). The group environment is set during the first session by the experienced clinical facilitators. The facilitator must establish credibility with the group by demonstrating cultural competency (e.g., talking about operational stress and compassion fatigue using terminology and examples commonly experienced by PSP). Facilitators begin establishing credibility during the first session, which helps to provide a space for vulnerability and sharing.

Second, BOS uses a group format comprised of other PSP to serve several functions. Many PSP occupations involve working in groups, which can make the BOS group format a fitting parallel. PSP report being more likely to trust friends and PSP colleagues (but not PSP leadership) than mental health professionals (Carleton et al., 2019b), suggesting the peer group may help provide a space for vulnerability and sharing. Group members can also become trusting and supportive of each other during their course of BOS. Participating in the group allows members to be mutually empowering based on (1) shared experiences; (2) learning coping strategies from other members; (3) developing a sense of belonging, acceptance, and security; and (4) healing through group disclosure (Yalom and Leszcz, 2005). The BOS program provides structural supports to maintain the group and therein potentiate benefits for an entire year, including developing strong bonds and continuing to act as peer supporters over the year-long program, which appears unique among current training programs. The BOS maintenance phase helps to solidify and reinforce the use of ideas presented during the first eight BOS content modules based on cognitive behavior therapy (Beck, 1976; Beck et al., 1985) as part of engaging with repeated PPTE exposures.

Third, BOS is designed to be proactive by promoting positive mental health habits. Without behavioral practice psychoeducation is unlikely to mitigate the impacts of PPTE exposures (Niles et al., 2012). BOS includes psychoeducational material but emphasizes and supports practical incorporation of the content over time within participants' regular environments. The BOS program is designed to shift participants' focus from adapting to adversity or PPTE exposures to developing skills that can be used proactively to protect mental health across a wide range of experiences. Skills can be implemented before and after PPTE exposures. Delivering BOS early in the career of many PSP may help to maximize the potential benefit for the individual (Robertson et al., 2015) and may help to support shifts toward increasingly psychologically safe PSP workspaces (Sivris and Leka, 2015). BOS participants who engage with program-recommended changes can help to develop broader organizational support, which can help to reduce self-reported stress (Maguen et al., 2009; Adams and Buck, 2010; Van der Velden et al., 2010); in addition, leadership support and organizational readiness for psychologically safe workspaces could contribute to successful adoption of programming for proactively protecting mental health (Knaak et al., 2019).

Fourth, BOS introduces functional disconnection and functional reconnection (FD/FR; McElheran and Stelnicki, 2021). There is an argument that effective physicians enter an “action mode,” setting aside emotional reactions and focusing on following protocol to prevent a patient's death (Whitehead, 2014, p. 273). Action mode appears similar to the mindset adopted by some PSP when conducting their duties. Functional disconnection may be argued by some as necessary for responding effectively to emergency calls and following procedures; in addition, functional disconnection aligns with the traditional stoic values of many PSP cultures (Sherman, 2007; Oliphant, 2016). Disconnection strategies can include shutting out emotional reactions of bystanders, visualizing the next technical step, and temporarily suppressing one's own emotional reaction to the situation (Regehr et al., 2002). McElheran and Stelnicki (2021) extend Whitehead's work by suggesting that functional *reconnection* may be a necessary sequalae to functional disconnection that occurs as the PSP transitions back into their personal life. Reconnection strategies include time for self-reflection or decompression after a shift (e.g., creating a demarcation point on the drive home where work is left and picked up during the next day's commute) and developing communication strategies with loved ones regarding the level of nervous system arousal before arriving home. Throughout the BOS program, PSP are taught skills that can help distinguish operational function from their personal lives and encouraged to develop consistent patterns of re-entry from work to home (Geller, 2017).

### Facilitation

BOS sessions are facilitated by licensed or registered master's or doctoral-level mental health clinicians (e.g., counselors, psychiatrists, psychologists, and social workers). The group is facilitated by one clinician if there will be fewer than seven participants or co-facilitated by two clinicians for groups of seven to 12 participants. All certified facilitators have completed a mandatory two-day training program provided by Dr. Megan McElheran and Dr. Milena Spasojevic of Wayfound Mental Health Group. Following the 2-day training, BOS clinicians must be supervised when facilitating their first two BOS groups. Supervision by an experienced BOS clinician is provided remotely or in-person, depending on the geographical location of the clinician and supervisor. Facilitators complete a fidelity checklist at the end of each session reporting on the extent to which the respective module content was covered. Facilitators also complete a rating of group cohesion. The fidelity check list and group cohesion report are used during supervision and to ensure consistent delivery across facilitators.

A significant component of the BOS clinician training program involves enhancing the cultural competency of clinicians who will be working with PSP. Most clinicians who have sought out BOS training have had at least some experience serving clients impacted by PPTE exposures and many have experience specifically with PSP or military populations. Clinicians who participated in the pilot BOS program underscored the importance of ensuring future facilitators understand the daily occupational experiences of PSP. Appreciating PSP experiences is likely critical for developing and maintaining rapport with PSP, which is crucial for participants' group engagement and maximization of potential benefits.

### Structure of the Program

BOS is a 16-h program divided into 8 weekly modules delivered in a group setting of up to 12 participants (10 participants is currently considered optimal). Each 2-h session is balanced between the presentation of didactic content (psychoeducation) and group processing time. The group processing allows participants to reflect on the presented psychoeducation materials and consider how to make the information personally relevant and applicable. Participants are encouraged to talk about the struggles and successes they have had relative to the psychoeducation content and generate ideas for ways to incorporate the psychoeducation content into their daily lives (i.e., homework). For example, during the third module (see below), participants turn their window of tolerance (Siegel, 2007) into a 10-point scale and then identify their own indicators of each level [e.g., levels 8–10 (hyper-arousal) can include panic, feeling overwhelmed, increased heart rate; levels 0–3 (hypo-arousal) can include numbing of emotions, disengagement from others]. Participants then monitor and chart their scale throughout the week. Following the fourth module, participants are asked to be on the lookout for cognitive distortions they experience. Even during the didactic portion, BOS is not delivered in a classroom-based lecture style; instead, facilitators are expected to engage the attendees.

Following the initial eight-session active phase of BOS, 10 monthly follow-up sessions are scheduled. The choice to include follow-up sessions came from client feedback regarding classroom-based programs (e.g., R2MR) that did not include follow-up or check-in sessions. Clients reported a lack of opportunity to implement knowledge or receive peer feedback on skills. Skill loss is known to occur without consistent practice or use (Arthur et al., 1998). The monthly sessions allow participants to implement skills in real life, as well as providing a set time to review, build, and develop BOS skills further. The time frame chosen is exploratory and may be adjusted following formal evaluation.

### Content of the Program

Each week facilitators briefly review previous content at the beginning of each session to maintain the connection between modules. The new module then builds upon the module before, reinforcing previous material and presenting new ways to cope with operational stressors during the active phase. Each of the eight modules has specific objectives:

- *Operational Service Culture*. The first session is designed to establish group expectations, and then define operational stress, OSIs, compassion fatigue, and moral injury. The session also provides a brief overview of the history of stoic service culture, when stoicism can be helpful, and when stoicism can be harmful. Functional disconnection and reconnection (FD/FR; McElheran and Stelnicki, 2021) is introduced as a mechanism whereby PSP can take an active role in transitioning between occupational and personal identities.
- *Physiology of Operational Stress*. The second session focuses on brain development with emphasis on how the stress response has evolved to support the survival of our species. The discussion centers on the autonomic nervous system, functions of the sympathetic and parasympathetic branches of the autonomic nervous system, hormonal dysregulation, and the impact that chronic stress can have on the brain.
- *Markers of Operational Stress*. The third session focuses on how unprocessed trauma can impact the nervous system, particularly in terms of dysregulation of the stress response, and describe OSIs as reflecting stress dysregulation. Participants learn how OSIs may manifest in hyper- or hypo-arousal of the nervous system as part of presenting the window of tolerance (Siegel, 2007), and then how to use the window of tolerance as a self-monitoring/self-awareness tool.
- *Cognitive Impacts*. The fourth session is designed to highlight the connections between thoughts, emotions, and behaviors through a discussion of automatic thoughts, cognitive distortions, and cognitive biases. Facilitators emphasize the importance of monitoring thoughts and ways to disrupt unhelpful thinking patterns when they are noticed.
- *Emotions*. The fifth session is designed to increase emotional awareness, describe the purpose of emotions, and help participants understand how emotions can help make sense of experiences. Cultural and societal influences on emotion are discussed, followed by how cultural expectations can lead to suppression of perceived unacceptable emotions.
- *Behavior*. The sixth session focuses on the role of avoidance in maintaining anxiety and post-PPTE exposure reactions, and how avoidance can interfere with the natural process of recovery. The concepts of habituation and *in vivo* exposure are introduced. Gradual exposure is proposed as a means of reducing problematic avoidance behaviors, with discussions of engagement strategies to increase perceptions of self-efficacy.
- *Communication*. The seventh session is designed to discuss how operational stress (both the stressor itself and the reaction to the stressors) can negatively impact communication. Key communication skills are presented to enhance interpersonal understanding.
- *Empathy and FD/FR*. The eighth and final session is designed to introduce how empathy can be helpful to the individual and the community they serve, but can also be a vulnerability during the PSP career. FD/FR is revisited as a strategy to balance the paradox of empathy. PSP are encouraged to recognize they can draw on different skills and values when engaged in occupational functions as compared to when they are engaged in their personal roles. Self-awareness is necessary to differentiate their roles through an intentional, actionable process. PSP are encouraged throughout the final module to identify specific strategies they will employ to facilitate FD/FR.

The group schedules 10 monthly meetings following the last module (i.e., Maintenance Phase). No specific content is scheduled for the monthly maintenance session. Instead, the maintenance sessions allow members opportunities for continued support from the group as well as ongoing opportunities for interactive problem-solving after the active training phase. The maintenance phase sessions are designed to further enhance integration of BOS training into members' working and personal lives. Facilitators support in-depth processing of ongoing challenges that members are experiencing as they incorporate BOS ideas into their daily routines. For example, one participant shared newly acquired ideas from the BOS group in the workplace and was criticized by peers for talking about mental health. The following session, the participant was able to ask for feedback about how other participants maintain a focus on self and stay healthy in a toxic work environment. The follow-up sessions also allow for ongoing benefits of group support, opportunities for self-reflection, and earlier support for referral to assessment or therapy where needed.

### Referral Criteria

BOS can be made available through individual or PSP organizational referral. A trained mental health clinician conducts a structured clinical intake interview with each potential member to ensure suitability for the group. The intake interview is completed shortly before the start of the program and includes discussion of the participant's experience with operational stress, previous treatment history, group expectations, informed consent, and current symptoms. Persons who are suffering from severe symptoms of any psychological condition that would interfere with the ability to tolerate being in a group (e.g., chronic PTSD symptoms), or experiencing active psychosis, are ineligible to participate in BOS.

### Purpose of the Study

The current study was designed to assess individual and group changes in several mental health symptoms commonly reported among PSP (i.e., depression, anxiety, stress, PTSD, and alcohol use), as well as changes in emotion regulation, guilt and shame, resilience, social support, and stigma from baseline (Time 1) to 10 months after the intervention (Time 6). Consistent with results from previous studies evaluating training programs delivered to PSP (e.g., Carleton et al., 2018b; Szeto et al., 2019), participation in BOS was hypothesized to result in small to moderate improvements in mental health symptoms, improve emotion regulation abilities, decrease feelings of shame and stigma, and enhance sense of personal resiliency and social support. Changes were expected to be sustained at each follow-up assessment, including at 1 year after the baseline.

## Methods

### Participants and Procedure

Participants were recruited *via* email following consent that was obtained during the intake interview to share their contact information with the researchers. Participants were emailed information about the study, a link to the consent form, and a link to the first survey for participation. BOS participants were told that participation in the program evaluation research was voluntary and would not impact their ability to continue in BOS or their ability to be referred for other mental health supports. BOS participants were also told that their BOS facilitator would not know which BOS group members participated in the research study. There were 203 BOS program registrants during the evaluation period and 155 (76.4%) expressed interest in participating in the research. Data was collected at six time points: pre-BOS (baseline; Time 1), post-BOS (at 2 months; Time 2), and at 1 month (Time 3), 4 months (Time 4), 7 months (Time 5), and 10 months (Time 6) post-BOS and immediately following the corresponding monthly maintenance session. Open-ended questions were included at the end of the Time 2 and Time 6 surveys to elicit qualitative data that would assist in understanding the participant's overall experience and perception of the program. The open-ended questions were also designed to obtain suggestions for improving the program components and delivery. The study was approved by the University of Regina Institutional Research Ethics Board (File #2018-191).

### Measures

#### Outcome Measures

Outcome measures were chosen based on the BOS module objectives. Each module has a specific topic focus, but concepts build throughout the program and are regularly revisited. BOS is designed to reduce active mental disorder symptoms and stress through awareness of cognitions, emotions, and behaviors across all sessions. Mental disorder symptoms were measured using well-validated tools, including the Depression Anxiety Stress Scale-21 (DASS-21; Lovibond and Lovibond, 1995). The DASS-21 contains three 7-item self-report scales to measure symptoms of depression, anxiety, and stress using a four-point Likert-type scale (0 = “did not apply to me at all”; 4 = “applied to me very much or most of the time”). Each scale (i.e., depression, anxiety, and stress) is totaled by summing the response totals and multiplying the score by two. The DASS-21 has strong convergent validity with other measures of anxiety and depression (Mitchell et al., 2008; Osman et al., 2012) and good internal consistency for each scale (Osman et al., 2012). Symptoms of PTSD over the past month were measured using the PTSD Checklist for DSM-5 (PCL-5; Weathers et al., 2013b). Participants first completed the Life Events Checklist-5 (LEC-5; Weathers et al., 2013a) and chose the PPTE they perceived as causing them the most current difficulty. Participants responded to 20 symptom items using a 5-point Likert-type scale (0 = “not at all”; 4 = “extremely”), with possible total scores ranging between 0 and 80. Previous psychometric analyses of the PCL-5 have evidenced strong test-retest reliability, and convergent and discriminant validity (Blevins et al., 2015; Ashbaugh et al., 2016). The Alcohol Use Disorders Identification Test (AUDIT; Babor et al., 2001) provided information about participants' alcohol use over the past year. The 10-item self-report questionnaire required participants to rate their alcohol use and related-behaviors on a 5-point Likert-type scale (0 = “never”; 4 = “daily or almost daily”). The AUDIT is a psychometrically valid and reliable screening tool, with strong test-retest reliability, sensitivity, and specificity (Reinert and Allen, 2002; Peng et al., 2012). For each mental disorder symptom measure, higher scores indicated higher levels or severity of symptoms.

Participants also completed measures of emotion regulation, guilt, shame, and stigma. Emotion regulation is the primary focus of the fifth module and appears informally throughout the program content. Emotional regulation was measured using the Difficulties in Emotion Regulation Scale (DERS; Gratz and Roemer, 2004), a 36-item self-report questionnaire designed to assess emotional dysregulation. Participants respond to each statement using a 5-point Likert-type scale (1 = “almost never”; 5 = “almost always”) and responses are summed. High scores indicated greater problems with emotion regulation. The DERS shows good internal consistency, construct validity, and predicts clinical severity and treatment outcome (Osborne et al., 2017; Hallion et al., 2018). Shame and stigma are discussed thoroughly in the first module, as well as during follow-up sessions as participants incorporate skills in real life. Participants' feelings of guilt and shame were measured using the Guilt and Shame Proneness Scale (GASP; Cohen et al., 2011). The GASP is a 16-item self-report scale measuring an individual's propensity to experience guilt and shame across several personal transgressions using a 7-point Likert-type scale (1 = “very unlikely”; 7 = “very likely”). It contains two Guilt subscales—Negative Behavior Evaluation (NBE; e.g., thinking “I made a mistake”) and Repair (e.g., apologizing)—and two Shame subscales—Negative Self Evaluation (NSE; e.g., thinking “I'm a terrible person”) and Withdraw (e.g., hiding)—with higher scores indicating higher levels of guilt and shame. The GASP requires more psychometric investigation, but appears to be a reliable and valid measure of guilt and shame (Cohen et al., 2011). Stigma was measured using the Avoidance and Danger/Unpredictable scales of the Opening Minds Survey for Workplace Attitudes (OMS-WA; Szeto et al., 2013). The 11-items were designed to measure attitudes about avoidance and danger/unpredictability toward people with mental illness. Each item was rated on a 5-point Likert-type scale (1 = “strongly disagree”; 5 = “strongly agree”), where higher scores indicated higher attitudes of stigma in the workplace. The OMS-WA is a standard metric for stigma used by the Mental Health Commission of Canada (Krakauer et al., 2020), although there is currently limited psychometric data available in the published literature.

Measures of perceived social support (Social Provisions Scale; Cutrona and Russell, 1987) and resiliency (Brief Resilience Scale; Smith et al., 2008) were also administered to BOS participants. The group format encourages peer support among group members and finding social support outside of the group. Resilience is an important variable to measure because of the proactive nature of the program. Cronbach's α for each scale for the current sample are in **Table 2**.

#### Qualitative Questions

There were five open-ended questions added to the survey at Time 2 and Time 6: (1) What has been the most helpful aspect of BOS for you?; (2) What has been the least helpful aspect of BOS for you?; (3) Has anything gotten better for you as a result of BOS? If so, please describe; (4) Has anything gotten worse for you as a result of BOS? If so, please describe; and (5) Please use the space below to provide any other comments you would like about your participation in BOS.

### Deviations From Proposed Procedure and Analysis

Several challenges arose during recruitment of participants that required amendments to the original proposed procedure and analysis. Research participation rates were relatively low and, consistent with most longitudinal research, participation declined across each time point (**Table 2**). Some participants did not complete consecutive surveys and there were large amounts of missing data. As such, we were only able to analyze changes up to Time 4. We were not able to analyze for group differences (e.g., by PSP category, by facilitator, by region) because of the small sample size and missing data. Qualitative data were obtained through open-ended survey questions at the end of the Time 2 and Time 6 surveys, rather than through focus groups. Group cohesion was assessed using the Therapeutic Factors Inventory (MacNair-Semands et al., 2010) and intended to be used as a moderator variable; however, the data did not allow for moderation analysis. Future analysis will be conducted as more data is collected.

### Analysis

First, descriptive analyses provided information about the frequencies and percentage of different PSP categories based on demographic variables. Next, means and standard deviations of outcome measures at each time point were calculated and plotted to illustrate changes over time.

Multilevel modeling (MLM) was performed to assess effects over time. Unbalanced longitudinal data sets (i.e., data sets including different numbers of observations per individual, or individuals measured at different time points) can be analyzed using MLMs [also known as linear mixed models (LMMs); Heck et al., 2013; West et al., 2015]. In the current study, we conducted a two-level model to assess the linear time trend. Applying MLM in our data set has several advantages; specifically, the results describe fixed effects, explain the source of variation at different levels, and provide more accurate estimations because of the missing values at different time points. Given the potential autocorrelation in longitudinal data, the first order autocorrelation, or AR (1), structure was also fitted to the within individual covariance. Restricted maximum likelihood (REML) was used as the estimation method in the current study that can provide more accurate results especially when sample size is small (Raudenbush and Bryk, 2002; Hox and de Leeuw, 2003). Standardized effect size (*ES*) estimates were calculated to assess the size of treatment effects over time. We interpreted the magnitude of effect size according to Cohen's *d* guidelines with 0.20 representing a small effect; 0.50 a medium effect; and 0.80 a large effect. Intraclass correlation (ICC) were used to measure the proportion of the variance due to differences between individuals.

The qualitative data were coded using NVivo 12 software. Data were coded inductively using descriptive codes (Miles et al., 2014). When relevant, data were coded to multiple codes to capture issues of overlap. When necessary, single survey responses containing multiple ideas were split to ensure multiple ideas were effectively captured. Data were coded thematically (as opposed to question-by-question) to identify themes across the dataset as a whole. The number of references as indicated below represents the total number of times a theme was mentioned, not the number of participants mentioning the theme. Therefore, in some cases, the reference number increased if a single participant mentioned the theme more than once; however, reference counts still serve as a useful indicator of importance. Reports of a specific number of participants referencing a given theme should be taken as the count of unique participants reporting the theme.

## Results

Sample characteristics are presented in Table 1. Participants were mainly men (48.5%) and were 30–39 years old (32.4%) or 40–49 years old (29.4%). PSP primarily reported being married or in common-law relationships (57.4%). Participants predominantly worked in Alberta (41.2%). Most PSP were firefighters (33.8%). Considerable proportions of participants had some post-secondary (42.6%) or a university (22.1%) education. There were 19 participants who completed the survey for all time points. The 19 participants were mainly married or common-law (68.4%), worked in Alberta (57.9%), and had a university degree (31.6%). There were 117 participants who did not complete the survey. The non-completers were predominantly men (49.6%), 30–39 years old (39), who were married or common-law (55.6%), working mainly in Alberta (38.5%), as firefighters (36.8%), with some postsecondary education (46.2%). Sample sizes, means, and standard deviations for all outcome measures are presented in Table 2.

**Table 1:** Public safety personnel demographics.

	**Total %** ** (*n*)^**a**^**	**Survey completer** ** % (*n*)^**b**^**	**Non completer** ** % (*n*)^**c**^**
**Gender**
Women	25.7 (66)	36.8 (7)	23.9 (28)
Men	48.5 (35)	42.1 (8)	49.6 (58)
**Age**
18–29	5.9 (8)	-	6.8 (8)
30–39	32.4 (44)	26.3 (5)	33.3 (39)
40–49	29.4 (40)	26.3 (5)	29.9 (35)
50–59	6.6 (9)	26.3 (5)	3.4 (4)
**Marital status**
Single	9.6 (13)	-	11.1 (13)
Married/Common-Law	57.4 (78)	68.4 (13)	55.6 (65)
Divorced/Separated/Widowed	5.9 (8)	5.3 (1)	6.0 (7)
**Province of residence**
Alberta	41.2 (56)	57.9 (11)	38.5 (45)
Ontario	26.5 (36)	-	30.8 (36)
Saskatchewan	6.6 (9)	21.1 (4)	4.3 (5)
**Education**
High school or less	7.4 (10)	15.8 (3)	6.0 (7)
Some postsecondary (less than 4-year college/university program)	42.6 (58)	21.1 (4)	46.2 (54)
University degree/4-year college or higher	22.1 (30)	31.6 (6)	20.5 (24)
**Public safety personnel category**
Firefighters	33.8 (46)	15.8 (3)	36.8 (43)
Paramedics	18.4 (25)	21.1 (4)	17.9 (21)
Municipal/provincial police	5.9 (8)	10.5 (2)	5.1 (6)
Royal Canadian Mounted Police	5.1 (7)	5.3 (1)	5.1 (6)
Crown prosecutors	8.1 (11)	15.8 (3)	6.8 (8)
**Total sample**	100 (136)	100 (19)	100 (117)

a *Total percentages may not sum to 100 and ns may not sum to 136 due to non-response or responding “other”.*

b *Total percentages may not sum to 100 and ns may not sum to 19 due to non-response or responding “other”.*

c *Total percentages may not sum to 100 and ns may not sum to 117 due to non-response or responding “other”*.

**Table 2:** Sample size and mean scores on outcome measures at each time point.

	**Cronbach's α**	**Time 1 (Baseline)**		**Time 2 (8 weeks)**		**Time 3 (3 months)**		**Time 4 (6 months)**	
		***n***	***Mean (SD)***	***n***	***Mean (SD)***	***n***	***Mean (SD)***	***n***	***Mean (SD)***
PTSD (PCL-5)	0.96	81	21.74 (16.87)	66	24.98 (18.17)	28	19.79 (13.90)	19	16.26 (13.79)
Depression (DASS-21 Dep)	0.89	74	8.51 (7.88)	64	9.53 (8.39)	29	7.93 (6.86)	19	6.74 (8.49)
Anxiety (DASS-21 Anx)	0.87	74	5.84 (7.30)	66	6.97 (6.72)	29	5.24 (5.03)	19	5.89 (7.23)
Stress (DASS-21 Str)	0.88	73	12.88 (8.77)	66	14.24 (8.60)	29	13.03 (8.94)	19	10.84 (7.87)
Alcohol Use Disorder (AUDIT)	0.54	63	7.27 (5.77)	57	7.12 (5.99)	24	5.08 (4.54)	15	7.60 (5.59)
Emotion Regulation (DERS)	0.95	72	81.10 (21.81)	65	85.34 (23.90)	28	77.86 (19.01)	19	72.32 (19.62)
Quality of Life (WHOQOL)	0.87	70	89.80 (13.30)	66	88.05 (12.22)	27	91.11 (10.55)	19	96.37 (12.19)
Social Support (SPS-10)	0.94	68	31.96 (5.73)	63	33.00 (4.53)	24	33.54 (5.12)	17	34.29 (4.38)
Stigma (OMSWA)	0.83	66	20.21 (5.71)	50	19.10 (5.92)	20	17.45 (4.68)	15	14.93 (5.79)
Guilt-Negative Behavior Evaluation (GASP)	0.74	64	23.34 (4.68)	48	23.71 (4.18)	20	24.20 (3.55)	15	24.20 (3.53)
Guilt-Repair (GASP)	0.57	64	22.88 (3.80)	48	22.60 (3.43)	20	23.50 (3.73)	15	23.13 (5.08)
Shame-Negative Self-Evaluation (GASP)	0.70	64	23.36 (4.44)	48	23.29 (4.14)	20	23.40 (4.01)	15	23.80 (3.57)
Shame-Withdraw (GASP)	0.59	64	11.13 (4.24)	48	11.25 (3.70)	20	10.30 (4.02)	15	9.33 (3.54)
Resilience (BRS)	0.92	70	3.54 (0.74)	66	3.41 (0.74)	28	3.68 (0.79)	19	3.71 (0.67)

*PTSD, Posttraumatic Stress Disorder; PCL-5, Posttraumatic Stress Disorder Checklist for DSM-5; DASS, Depression, Anxiety, and Stress Scale; AUDIT, Alcohol Use Disorders Identification Test; DERS, Difficulties in Emotion Regulation Scale; WHOQOL, World Health Organization Quality of Life; SPS-10, Social Provisions Scale; OMSWA, Opening Minds Survey for Workplace Attitudes; GASP, Guilt and Shame Proneness Scale; BRS, Brief Resilience Scale*.

### Symptom Changes

**Figure 1:**
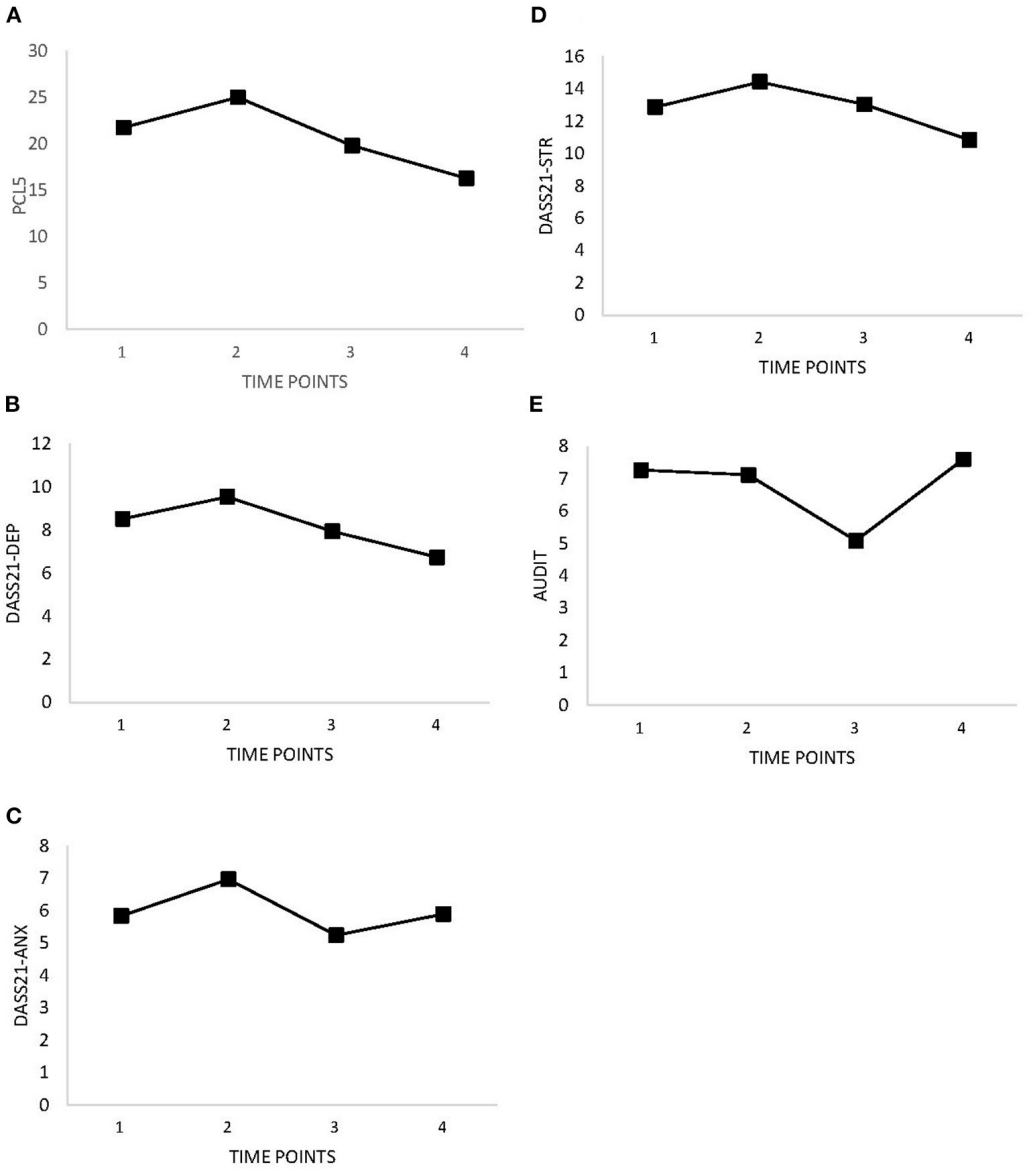
Changes in self-reported mental disorder symptoms as measured at baseline (Time 1), 8 weeks (Time 2), 3 months (Time 3), and 6 months (Time 4). **(A)** PTSD (PCL-5). **(B)** Depression (DASS-21). **(C)** Anxiety (DASS-21). **(D)** Stress (DASS-21). **(E)** Alcohol Use Disorder (AUDIT).

**Figure 2:**
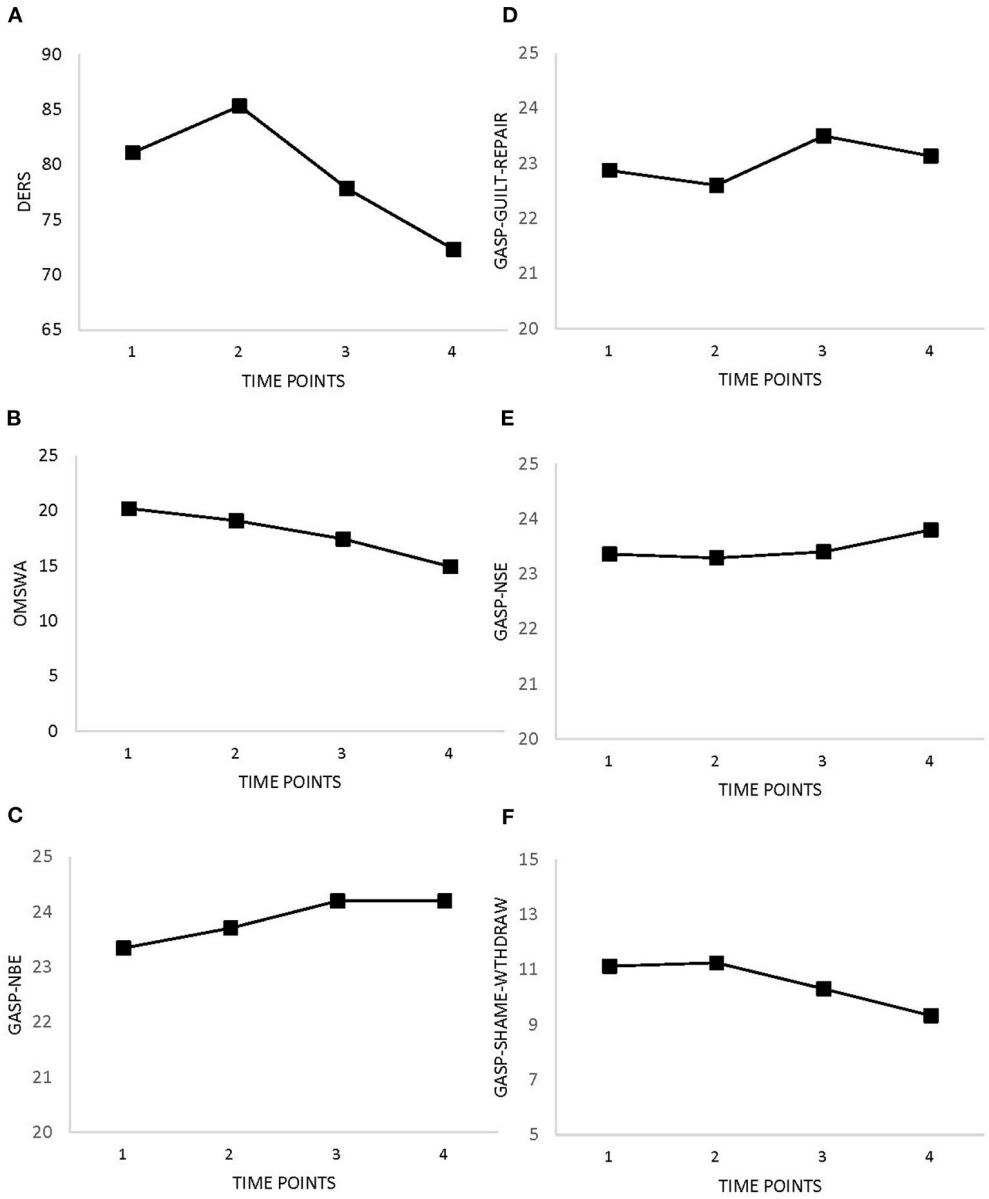
Changes in self-reported emotion regulation, stigma, guilt and shame as measured at baseline (Time 1), 8 weeks (Time 2), 3 months (Time 3), and 6 months (Time 4). **(A)** Emotion Regulation (DERS). **(B)** Stigma (OMSWA). **(C)** Guilt-Negative Behavior Evaluation (GASP). **(D)** Guilt-Repair (GASP). **(E)** Shame-Negative Self-Evaluation (GASP). **(F)** Shame-Withdraw (GASP).

**Figure 3:**
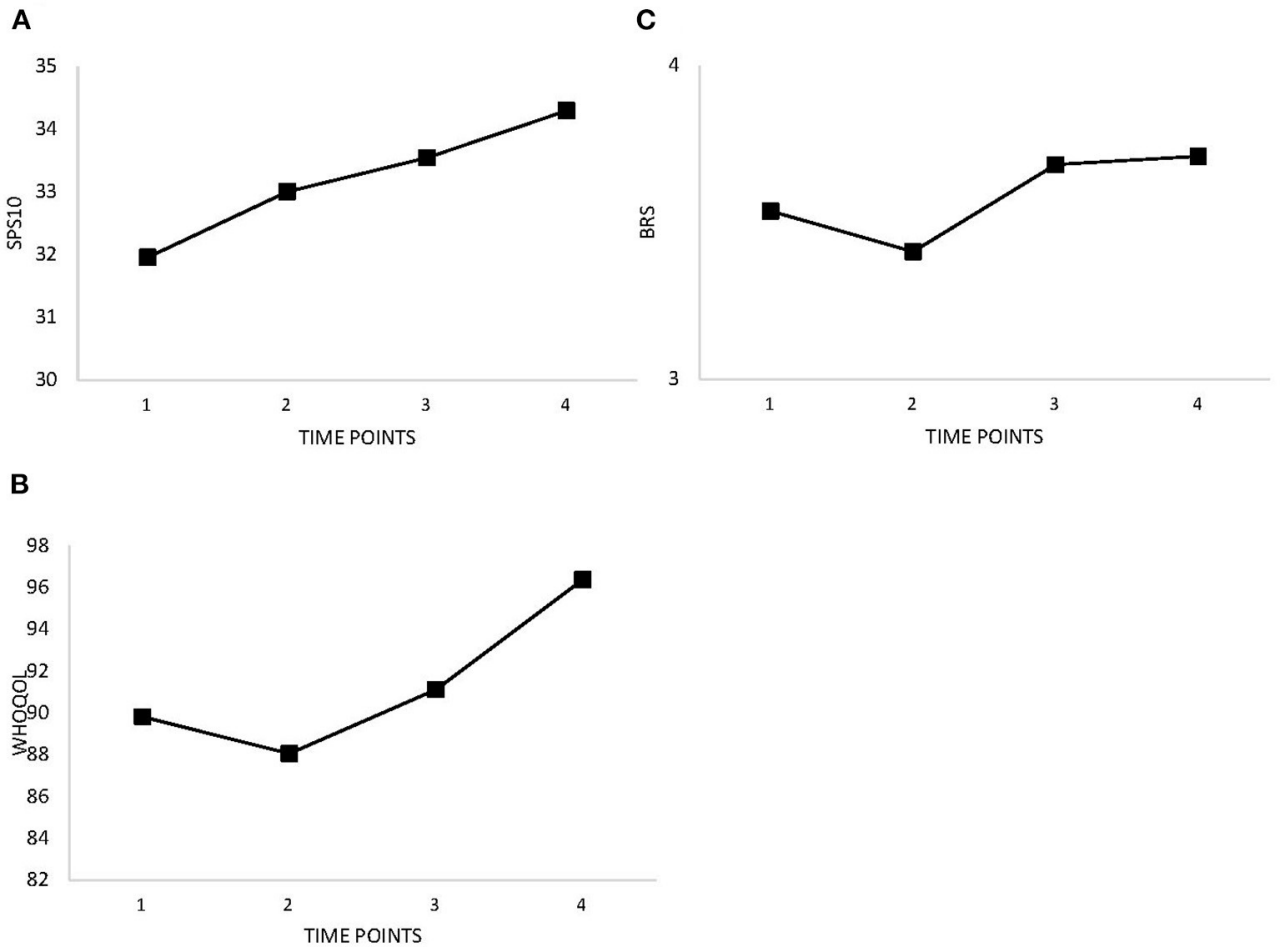
Changes in social support, resilience, and quality of life as measured at baseline (Time 1), 8 weeks (Time 2), 3 months (Time 3), and 6 months (Time 4). **(A)** Social Support (SPS-10). **(B)** Quality of Life (WHOQOL). **(C)** Resilience (BRS).

Table 2 and Figure 1 show improvements in symptoms of PTSD, depression, anxiety, stress, and alcohol use over time. There were slight increases between Time 1 (baseline) and Time 2 (after completion of BOS). Improvements (i.e., decreases in measure scores) were expected for emotion regulation, guilt and shame proneness, and stigma. Table 2 and Figure 2 show decreasing trends for each measure except guilt, which showed slight increases. Improvements (i.e., increases in measure scores) were expected for social support, quality of life, and resilience. Overall, increases were observed for perceived social support, quality of life, and resilience (Table 2 and Figure 3); however, slight decreases were observed for quality of life and resilience from Time 1 to Time 2.

Consistent with the patterns in Table 2, the mental disorder symptom measure scores decreased over time, whereas quality of life and perceived social support increased (see Average Growth Effect in Table 3). There were statistically significant changes only for PTSD (γ = −1.84, *p* <0.05), quality of life (γ = 1.11, *p* < 0.05), perceived social support (γ = 0.78, *p* < 0.05) and stigma (γ = −1.01, *p* < 0.01) through Time 4. The γ's values represent the average change over time across all individuals. The current results imply that participation in the BOS program produced small, yet statistically significant improvements in PTSD symptoms (*ES* = 0.11), quality of life (*ES* = 0.09), perceived social support (*ES* = 0.17), and stigma (*ES* = 0.18). The two random effects (i.e., between-person variance at baseline level and within-person variance) were statistically significant, indicating a statistically significant amount of variation in outcome measures at different time points was due to differences between individuals, as well as differences within each person across different time points. The ICC (Table 3) represents the magnitude of variance in outcome measures due to variation between individuals. For example, 69.9% of variation in PTSD was due to differences between individuals rather than differences over time; thus, up to 30.1% of the variation in PTSD was related to differences over time and measurement error.

**Table 3:** Two-level mixed model estimates for outcome measures.

	**Fixed effects**		**Random effects**		**ICC**	**Effect size (*d*)**
	**Average baseline level (γ_00_)**	**Average growth effect (γ_10_)**	**Between person variance in baseline level (τ_00_)**	**Within individual variance (σ^2^)**		
	**Estimate (SE), [CI]**	**Estimate (SE), [CI]**	**Estimate (SE), [CI]**	**Estimate (SE), [CI]**		
PTSD (PCL-5)	23.64 (1.63)^***^, [20.41, 26.86]	−1.84 (0.81)^*^, [−3.46, −0.21]	201.88 (36.12)^***^, [−0.63, 0.02]	88.17 (14.73)^***^, [63.56, 122.29]	0.696	0.11
Depression (DASS-21 Dep)	9.09 (0.81)^***^, [7.49, 10.69]	−0.73 (0.38), [−1.49, 0.04]	48.28 (8.78)^***^, [33.81, 68.95]	16.64 (3.44)^***^, [11.10,24.94]	0.743	0.09
Anxiety (DASS-21 Anx)	6.51 (0.69)^***^, [5.15, 7.88]	−0.19 (0.35), [−0.90, 0.52]	31.67 (6.28)^***^, [21.47, 46.70]	15.02 (3.04)^***^, [10.11, 22.33]	0.678	0.03
Stress (DASS-21 Str)	13.95 (0.90)^***^, [12.18, 15.72]	−0.59 (0.48), [−1.55, 0.38]	47.86 (11.14)^***^, [30.33, 75.54]	28.12 (6.76)^***^, [17.56, 45.05]	0.630	0.07
Alcohol Use Disorder (AUDIT)	7.50 (0.61)^***^, [6.30, 8.70]	−0.38 (0.20), [−0.80, 0.03]	30.77 (4.88)^***^, [22.55, 41.99]	3.13 (0.94)^***^, [1.74, 5.65]	0.908	0.07
Emotion Regulation (DERS)	83.28 (2.26)^***^, [78.81, 87.74]	−1.53 (1.02), [−3.58, 0.52]	373.21 (75.50)^***^, [251.05, 554.82]	121.72 (40.91)^***^, [62.99, 235.22]	0.754	0.07
Quality of Life (WHOQOL)	88.49 (1.27)^***^, [85.98, 91.01]	1.11 (0.53)*, [0.04, 2.18]	128.84 (21.64)^***^, [92.71, 179.05]	29.78 (6.91)^***^, [18.90, 46.94]	0.812	0.09
Social Support (SPS-10)	31.98 (0.55)^***^, [30.91, 33.06]	0.78 (0.33)^*^, [0.13, 1.43]	-	22.34 (3.25)^***^, [16.81, 29.70]	-	0.17
Stigma (OMSWA)	19.79 (0.61)^***^, [18.58, 21.00]	−1.01 (0.31)^**^, [−1.64, −0.38]	25.35 (4.72)^***^, [17.61, 36.51]	6.77 (1.87)^***^, [3.94, 11.64]	0.789	0.18
Guilt-Negative Behavior Evaluation (GASP)	23.49 (0.44)^***^, [22.69, 24.38]	0.05 (0.21), [−0.40, 0.49]	12.43 (2.38)^***^, [−0.97, −0.73]	6.20 (1.43)^***^, [3.94, 9.75]	0.667	0.01
Guilt-Repair (GASP)	22.79 (0.38)^***^, [22.02, 23.55]	0.15 (0.30), [−0.46, 0.76]	7.94 (1.81)^***^, [5.09, 12.40]	4.27 (0.95)^***^, [2.76, 6.61]	0.651	0.04
Shame-Negative Self-Evaluation (GASP)	23.28 (0.48)^***^, [22.34, 24.23]	0.07 (0.32), [−0.58, 0.71]	-	14.18 (2.16)^***^, [10.51, 19.13]	-	0.02
Shame-Withdraw (GASP)	11.08 (0.42)^***^, [10.24, 11.92]	−0.07 (0.17), [−0.42, 0.28]	12.41 (2.21)^***^, [8.75, 17.60]	3.71 (0.78)^***^, [2.46, 5.59]	0.770	0.02
Resilience (BRS)	3.48 (0.08)^***^, [3.33, 3.63]	0.01 (0.04), [−0.06, 0.08]	0.42 (0.07)^***^, [0.29, 0.59]	0.15 (0.03)^***^, [0.11, 0.21]	0.736	0.01

* *p < 0.05,*

** *p < 0.01,*

*** *p < 0.001.*

SE, Standard error; CI, 95% Confidence interval; ICC, intraclass correlation; PTSD, Posttraumatic Stress Disorder; PCL-5, Posttraumatic Stress Disorder Checklist for DSM-5; DASS, Depression, Anxiety, and Stress Scale; AUDIT, Alcohol Use Disorders Identification Test; DERS, Difficulties in Emotion Regulation Scale; WHOQOL, World Health Organization Quality of Life; SPS-10, Social Provisions Scale; OMSWA, Opening Minds Survey for Workplace Attitudes; GASP, Guilt and Shame Proneness Scale; BRS, Brief Resilience Scale.

a *This covariance parameter is redundant. The test statistic and confidence interval cannot be computed*.

### Exploratory Analysis: Qualitative Results

The qualitative data indicated that participants' views of the BOS program were primarily positive. All respondents noted that the program had been helpful or beneficial to them in some way. Many participants skipped the question about the least helpful aspects of BOS or used the opportunity to state that there was nothing they disliked. One participant commented, “I didn't realize how much I really needed it,” and another said, “Overall, BOS has changed so many aspects of my life in a positive way that I cannot decide on a part that was not helpful.”

There were 28 references and the most frequently coded theme was “Understand / Recognize Feelings or Mental State”. Participants often spoke of how the BOS program had enhanced their awareness of their own emotions: “[I am] better at monitoring my feelings throughout the day and dealing with them.” Another participant linked their enhanced awareness to the possibility of increased resiliency: “I have found it helpful in developing emotional insight and (hopefully) some additional resiliency.” For other participants, the increased awareness of emotions was difficult. For one participant, the program had prompted “Self-realization, which is tough.” Several participants noted that the program had aggravated their symptoms or revived memories of past PPTE exposures. Although sometimes seen as a negative aspect of participation, most participants noted that such short-term pain was necessary for healing in the long term. One said: “I feel things are worse but that is because I am more aware and educated about my feelings and thoughts. I am worse but that's due to less avoidance, which is better in the long run.”

In many cases the enhanced awareness was associated with identifiable behavioral changes (13 references). Several participants had sought out additional psychological supports after completing the program: “Yes, it has triggered traumatic stress; however, I am now getting help after being assessed for PTSI [posttraumatic stress injury]. I am fortunate to have access to help.” The results suggest the importance of ensuring that subsequent psychological support is available to those who complete the program. Other behavioral changes included: better coping, more effective response to stressful situations, ability to recognize the emotions of others and better support others, and increased prevention and resiliency. One participant noted that, “I have been avoiding arguments when I am hyper-aroused and so far so good.”

The reported benefits appeared to extend to participants' family and work lives. There were six participants who mentioned benefits to their relationships with partners or children. “I am better toward my family,” one noted. There were two participants who reported better relationships with colleagues, including a “tighter bond” and “more support from colleagues,” and a third identified a generally improved work life. The improved relationships were likely connected, in part, to more effective communication practices—another reported outcome of the BOS program—with five total references.

After increased emotional awareness, the second most frequently coded theme was “Feeling Normal/Not Alone” (13 references). Participants often spoke about the benefits of feeling connected to others with a similar experience. Participants reported appreciating “knowing I'm not alone in how I feel,” “realizing the rest of the group has similar issues,” and “realizing that my issues are common amongst my peers.” In a few cases, knowledge of the science behind OSIs was helpful and contributed to the feeling of normalcy. There was a general appreciation for the educational component, including access to psychologists, psychological and biological data, and other scientific information.

In contrast to the benefits reported from BOS content, views of the group component were mixed. Some participants reported believing they would have been “more open” in a one-on-one setting. Disclosure was a challenge for some participants, who reported a discomfort with group members who over-shared personal stories or a dislike for “airing personal aspects of home life.” There were two participants who reported not enjoying the virtual online sessions, which they felt were less effective or desirable. There was one participant who also suggested more content on physiological aspects of OSIs would help, which reflects the previously mentioned benefits of understanding the science behind mental health problems.

BOS tends to facilitate participants experiencing potentially challenging emotions or recalling past PPTE exposures; as such, there was one participant suggested placing the coping skills early in the program. Several participants reported wanting to see the group move to biweekly meetings rather than monthly, as the frequency was preferred. Some participants reported having difficulties finding time away from work to complete the program, but participants overwhelmingly described the program as worthwhile. Timing was considered an important area for future change; specifically, several participants suggested the importance of attending the program early in one's career. There was one participant who self-identified as an “old dog” who described “the BOS program is integral for every new recruit, prior to operational stress.”

## Discussion

The current study was designed to evaluate the Before Operational Stress (BOS) program, an evidence-informed program to proactively support the psychological health of PSP. Overall, participants' perceptions of the program were positive. As expected, improvements in symptoms of PTSD, depression, anxiety, and stress, and alcohol use over time were observed in participants who attended the program. Similarly, improvements were observed in measures of quality of life, perceived social support, resilience, emotional regulation, and stigma. Small, statistically significant changes were only found for improvements in PTSD, quality of life, perceived social support, and stigma from Time 1 to Time 4.

There have been few evaluations of mental health training programs for PSP (Beshai and Carleton, 2016; McCreary, 2019; Anderson et al., 2020). The current study contributes to the limited published peer-reviewed literature. The BOS program appears to result in decreased stigma for 4 months following program completion and produced a statistically significant reduction in PTSD symptoms through 6 months post-baseline.

Slight increases in mental disorder symptoms and decreases in ratings of quality of life and resilience occurred from Time 1 and Time 2. The worsening of symptoms and associated factors may be explained in part by increases in participants' self-awareness by the end of the 8-week dyadic component. For example, one participant reported feeling things were worse because they were more aware of their emotional states; however, the same participant also reported no longer avoiding unpleasant feelings and thoughts, which was identified as an important long-term. Self-awareness can clarify a person's attitudes, sensations, and emotions, leading to an amplification of negative affect (Carver, 1975; Scheier and Carver, 1977; Silvia, 2002). Alternatively, the poorer symptoms and associated factors may have been related to the weekly group meetings ending, causing a loss of consistent social support, and participants' becoming concerned about managing their symptoms without help. Contrasting expectations, the guilt subscales of the GASP showed increasing trends over time, whereas the shame subscales showed expected evidence of decreasing over time, but neither set of changes were statistically significant. Ongoing analyses should clarify the impact of the BOS program on feelings of guilt and shame.

The observed small effect sizes in the outcome measures may have been due to the unusually large amount of individual variability. For example, almost 70% of variation in PTSD was due to differences between individuals, suggesting about 30% of the variation in PTSD symptom changes was related to differences over time and measurement error. We were unable to determine how much of the change was attributed to measurement error. One explanation for the large individual variability was the lack of homogeneity within the groups; groups contained PSP from various sectors with different levels and presentations of the symptoms measured. Some participants may be entering the group with clinically significant symptom levels, whereas others may have attended the group with more proactive intentions. Future analyses could separate participants into clinical and non-clinical groups based on their self-ratings at Time 1 to determine program effectiveness for different symptom profiles, and to avoid floor and ceiling effects.

Participant recommendations for program modifications included: (1) ensuring that psychological support is available to those who complete the program; (2) offering different delivery modalities (e.g., online or in person; some participants preferred in-person meetings whereas others preferred virtual meetings); (3) introducing coping skills earlier in the program; (4) biweekly maintenance sessions (instead of monthly meetings); (5) emphasizing participation in the program early in PSP careers; and (6) having the program offered at varied times to accommodate PSP shift work. The exploratory qualitative analysis suggested that the improvements that participants attributed to BOS may not have been adequately captured by the outcome measures used in the evaluation. For example, participants reported changes in self-awareness and self-monitoring of feelings and thoughts; behavioral changes (e.g., better coping and response to stressful situations; recognizing when others need support; more preventative actions); and improved relationships with family and colleagues. The outcomes identified for BOS were not directly measured as part of the current evaluation. Inclusion of such measures in ongoing evaluations of the program may be beneficial in highlighting potential benefits of attending the BOS program.

### Limitations and Directions for Future Research

The current study results contribute to a large gap in understanding the effectiveness of programs for PSP, and the current limitations provide important directions for future research. A significant study limitation is participant attrition. Research participation was not mandatory for BOS registrants and 155 of 203 (76.4%) registrants expressed interest in research participation; however, only 81 participants completed the survey at Time 1, 66 completed the survey at Time 2, 29 completed the survey at Time 3, and 19 completed the survey at Time 4. Survey responses were also inconsistent; for example, some participants completed the first survey and one other survey throughout the data collection period resulting in incomplete data sets for participants. We are unable to assess why participants did not complete certain studies, but future studies investigating demographic variables associated with non-completion (see Table 1) appear warranted. Previous literature has showed participants who are younger and have lower education levels are more likely to not complete longitudinal studies (de Graaf et al., 2013); it is unlikely that mental disorders and clinical characteristics prevented participants from completing the study (de Graaf et al., 2000, 2013). Due to the small sample size, we have not been able to analyze the results to assess for differences based on demographics variables such as PSP sector category (e.g., paramedics relative to firefighters), participant gender, facilitator fidelity, group cohesion or geographic region. Participants were also active-duty PSP, even though BOS would ideally be provided prior to or early in the PSP's career. A longitudinal randomized controlled trial with early-career PSP would provide the strongest evidence for use of the program. Additionally, the COVID-19 pandemic led to some BOS groups completing their BOS training or maintenance virtually instead of in person, and we are unsure impact virtual delivery had on outcome improvements. Data will continue to be collected as BOS training is delivered, and future analyses will compare delivery modalities (i.e., virtual versus in person) and variation in maintenance sessions (e.g., no maintenance or fewer maintenance sessions). Wayfound Mental Health Group will also implement a train-the-trainer program in the near future, allowing for wider availability. More extensive evaluation of the program is necessary before the BOS program can be considered an evidence-based program for PSP; however, the initial results suggesting small improvements in symptoms of PTSD, quality of life, perceived social support, and stigma are promising.

## Conclusions

PSP are at risk of developing mental health problems. Participation in proactive programs focused on protecting PSP mental health may reduce the risk and encourage early help-seeking. The BOS program is a clinically- and evidence-informed 8-week, group-based mental health training program combining theoretical and experiential learning to mitigate the impacts of operational stress. BOS is also delivered by PSP culturally competent facilitators who have received special training and have expertise in working within the context of PSP environments.

Early evidence suggests that participation in the BOS program supports small, statistically significant improvement in PTSD symptoms, quality of life, perceived social support, and stigma until at least 6 months from baseline. Qualitative responses revealed other areas of improvement, including self-awareness, behavioral changes, and improved relationships with family and colleagues, which were not measured quantitatively. Further evaluation and research are needed to better understand how long outcomes are sustained, and to assess for sociodemographic differences. BOS appears to be a promising training program for the proactive protection of PSP mental health and is one of few such programs with any empirical support. All training programs intended to provide proactive protection for PSP mental health must develop an empirical base to support widespread adoption, continuous improvement, and culturally-appropriate options for clinicians working with PSP.

## Data Availability Statement

The datasets presented in this article are not readily available because of potentially identifiable information that could compromise participant confidentiality. Requests to access the datasets should be directed to andrea.stelnicki@uregina.ca.

## Ethics Statement

The studies involving human participants were reviewed and approved by University of Regina Institutional Research Ethics Board (File #2018-191). The patients/participants provided their written informed consent to participate in this study.

## Author Contributions

AS and RNC managed research design, recruitment, and data collection. AS, LJ, and AF were responsible for data analysis. All authors contributed to the first manuscript draft, manuscript revision, and approved the submitted version.

## Conflict of Interest

The authors declare that the research was conducted in the absence of any commercial or financial relationships that could be construed as a potential conflict of interest.

## Publisher's Note

All claims expressed in this article are solely those of the authors and do not necessarily represent those of their affiliated organizations, or those of the publisher, the editors and the reviewers. Any product that may be evaluated in this article, or claim that may be made by its manufacturer, is not guaranteed or endorsed by the publisher.

## Footnotes

^1^WGM Psychological Services is now Wayfound Mental Health Group. Wayfound Mental Health Group is a private psychological practice with locations in Alberta and Ontario, Canada. (www.wayfound.ca).

## Abbreviations

AUDIT: Alcohol Use Disorders Identification Test
BOS: Before Operational Stress
CIPSRT: Canadian Institution for Public Safety Research and Treatment
DASS-21: Depression Anxiety Stress Scale-21
DERS: Difficulties in Emotion Regulation Scale
DSM: Diagnostic and Statistical Manual of Mental Disorders
ES: Effect size
FD/FR: Functional disconnection/functional reconnection
GASP: Guilt and Shame Proneness Scale
ICC: Intraclass correlation
LEC-5: Life Events Checklist-5
LMM: Linear mixed model
MLM: Multilevel modeling
OMS-WA: Opening Minds Survey for Workplace Attitudes
OSI: Operational stress injury
PCL-5: PTSD Checklist for DSM-5
PPTE: Potentially psychologically traumatic events
PSP: Public Safety Personnel
PTSD: Posttraumatic stress disorder
PTSI: Posttraumatic stress injury
R2MR: Road to Mental Readiness
REML: Restricted maximum likelihood
WWC: Wounded Warriors Canada.
